# Heteroatom Engineering in Robust Al-Based MOFs for Efficient Separation of Xenon over Krypton

**DOI:** 10.3390/molecules31050891

**Published:** 2026-03-07

**Authors:** He Wang, Zhiyan Zhang, Yingying Xu, Yang Lu, Ying Tian, Guangjie Zhang, Sifan Liu, Shuchen Liu

**Affiliations:** 1 College of Chemistry and Materials Science, Hebei University, Baoding 071002, China; w543051071@163.com; 2 Beijing Institute of Radiation Medicine, Beijing 100850, China; zzyan0911@163.com (Z.Z.); 2445010989@stu.ahmu.edu.cn (Y.X.); 19832267208@163.com (Y.L.); tianying1977@126.com (Y.T.); 3 College of Life Sciences, Hebei University, Baoding 071002, China; 4 School of Pharmacy, Anhui Medical University, Hefei 230032, China

**Keywords:** metal–organic frameworks, xenon/krypton separation, heteroatom engineering, aluminum-based MOFs

## Abstract

The separation of xenon (Xe) and krypton (Kr) is very important for industrial applications and environmental protection. However, the lack of permanent dipoles, low polarizabilities arising from their spherical nature, and similar kinetic diameters make their efficient separation by porous adsorbents exceptionally challenging. This study explored the effects of pore geometry and surface polarity of a series of aluminum-based metal–organic frameworks (CAU-10-H, MIL-160, KMF-1, CAU-23) on Xe/Kr separation performance using a heteroatom engineering strategy. These MOFs are composed of AlO_6_ clusters and bent dicarboxylic acid linkers, enabling us to systematically investigate the effects of pore size and heteroatom types on Xe/Kr separation performance. Among them, MIL-160 has a polar linker based on furan, showing the best balance performance. At 298 K and 1.0 bar, the uptake of Xe is 4.12 mmol g^−1^ and the IAST selectivity is 7.63 for a Xe/Kr (20/80) mixture. The practical performance was verified by dynamic breakthrough experiments, which yielded a long Xe breakthrough time of 42.9 min g^−1^. Grand Canonical Monte Carlo (GCMC) simulations and first-principles density functional theory (DFT) calculations revealed that the enhanced performance originates from cooperative confinement and polarization effects, with the furanyl oxygen atoms providing optimal Xe-binding sites. This work clarifies the structure–property relationships governing Xe/Kr separation in aluminum-based MOFs (Al-MOFs), highlighting the potential of heteroatom engineering for designing efficient noble gas adsorbents.

## 1. Introduction

High-purity xenon (Xe) and krypton (Kr) are indispensable due to their wide applications, including anesthesia [1,2], gas lasers [3,4], medical imaging [5], commercial lighting [6] and aerospace technologies [7]. However, the extremely low concentrations of Xe (0.087 ppmv) and Kr (1.14 ppmv) in the atmosphere poses a significant challenge to separate these essential gases from air [8]. A Xe/Kr mixture (20/80, *v*/*v*) is obtained as a byproduct of the air separation industry. Pure Xe and Kr gases are subsequently separated through cryogenic distillation [9]. Furthermore, the low-concentration ^127^Xe, ^133^Xe, ^135^Xe and ^85^Kr isotopes are released into the atmosphere during reactor operation of the nuclear industry [10,11] and can enter the atmosphere during the reprocessing of used nuclear fuel (UNF) [12]. The significant disparity in half-life between ^85^Kr (~10.8 years) and ^127^Xe (~36 days) necessitates their highly efficient separation, which is imperative to prevent the uncontrolled release and enable the beneficial reuse of xenon [13]. Although cryogenic distillation is a conventional approach that exploits differences in boiling point for Xe/Kr separation, it is highly energy-intensive and capital-intensive [14]. Thus, there is a strong need to develop efficient, low-cost, and mild separation technologies as energy-efficient alternatives. However, the inert gases Xe and Kr lack both dipole and quadrupole moments, have very similar kinetic diameters (Xe: 4.047 Å; Kr: 3.655 Å), and exhibit low polarizabilities (Xe: 4.04 × 10^−25^ cm^3^; Kr: 2.48 × 10^−25^ cm^3^) [15]. Consequently, the separation of Xe/Kr mixtures remains an extreme challenge. Adsorptive separation based on solid porous adsorbents appears to be a promising alternative to the cryogenic distillation process because of lower energy consumption and milder operating conditions [16,17]. Many traditional solid adsorbents, such as activated carbons [18,19,20] and molecular sieves [21,22,23], have been extensively investigated for gas separation. Notably, metal–organic frameworks (MOFs), as a new type of porous material constructed by metal clusters and organic linkers, have exhibited distinctive advantages due to their exceptional tunability on pore structures and pore chemistry.

The strategic incorporation of polar functional sites such as halogens, hydroxyl groups, and nitrogen-containing moieties into metal–organic frameworks provide a powerful avenue for enhancing Xe selectivity and adsorption capacity in Xe/Kr separation. A notable example is the squarate-based MOF 1a ([Co_3_(C_4_O_4_)_2_(OH)_2_]), which incorporates polar –OH groups and achieves a Xe/Kr Henry selectivity of 54.1 [24]. Early studies on IRMOF-2-X series (X = F, Cl, Br, I) have shown that increasing halogen polarizability through hydroxyl decoration can enhance Xe uptake in MOFs [25]. This principle has been advanced in the more recent studies on LIFM-DMOF-X1 [26] and LIFM-DMOF-X2 [27] families, where halogen identity is explicitly shown to simultaneously modulate pore aperture and surface polarity, thereby synergistically boosting Xe affinity. Furthermore, nitrogen-containing functional groups, such as the –NH_2_ group in Mg-SBMOF-1, significantly enhance Kr uptake by increasing ligand polarizability [28]. In parallel with the study of polar sites, there is a clear research impetus to address other critical determinants of performance, such as the exact pore size matching with Xe [29] and the necessity for hydrolytic and thermal stability in practical applications [30,31]. Therefore, Al-MOFs have become a promising research platform due to the exceptional physicochemical stability originating from strong Al–O bonds. The inherent robustness, coupled with characteristics such as low density and potential for aqueous synthesis [32], inspired the study of a series of Al-MOFs for Xe/Kr separation. Notable examples are MIL-120 [30], CAU-10-H-CH3-0.21–0.79 [33], Al-SDB [34], ZUL-C2 [31], Al-CDC [35] and Ag-MOF-303 [36].

Based on the significance of polar functional sites, this work systematically investigates the influence of different heteroatoms (N, O, S) within the pore channels of robust Al-MOFs on Xe/Kr separation. Three water-stable, cost-effective aluminum-based MOFs, KMF-1, MIL-160, and CAU-23, were studied in this research, which were constructed from heterocyclic dicarboxylate ligands with N, O, and S heteroatoms, respectively. Comparatively, another Al-MOF without heteroatoms, CAU-10-H, was also synthesized to provide a reference pore surface with distinct polarity. While the topological structures are not completely identical, their frameworks share a similar building block of AlO_6_ chains, providing a stable platform to correlate heteroatom identity with adsorption performance. The separation potential was evaluated through single-component Xe/Kr adsorption isotherms and dynamic breakthrough experiments. Although the overall Xe uptake and selectivity values are comparable, the subtle differences observed are critically analyzed and linked to their distinct pore surface chemistries. Combined with molecular simulations, this study reveals how the specific heteroatom modulates the Xe–framework interaction, providing fundamental insights for the precise engineering of pore environments in stable MOFs for advanced gas separations.

## 2. Results and Discussion

### 2.1. Analysis of Structural Characterization

**Figure 1:**
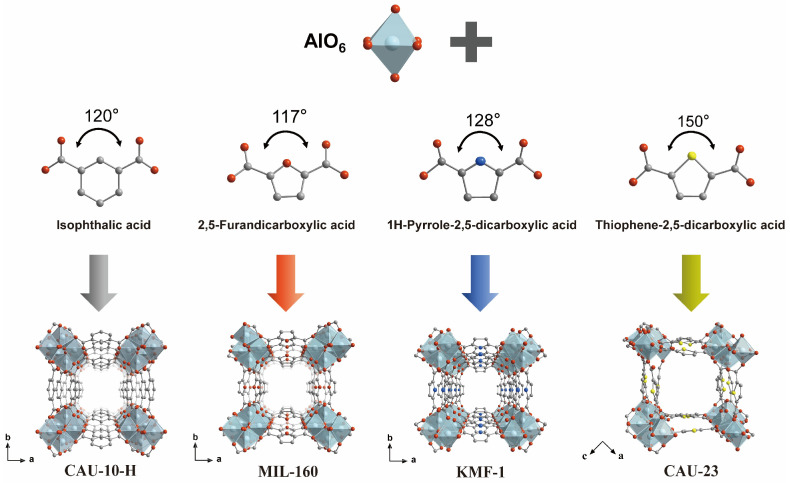
Schematic diagram of crystal synthesis of CAU-10-H, MIL-160, KMF-1 and CAU-23. The color scheme designates Al, C, N, O, and S in blue, gray, dark blue, red, and yellow, respectively. CIF: 2323179 (CAU-10-H); 2219217 (MIL-160); 1984701 (KMF-1); 1878820 (CAU-23).

The tested Al-MOFs (CAU-10-H, MIL-160, KMF-1, CAU-23) are constructed from rod-shaped secondary building units (SBUs) composed of corner-sharing AlO_6_ octahedra infinite chains, where each octahedral Al^3+^ center is fully coordinated by four carboxylate oxygen atoms from organic linkers and two bridging μ-OH groups. This common architecture dictates their one-dimensionally (1D) confined channel structures (Figure 1). However, this common structure is cleverly transferred into a unique topology and pore structure by organic linkers. For CAU-10-H, MIL-160, and KMF-1, the cis-corner-sharing AlO_6_ chains are connected by bent dicarboxylic acid ligands (isophthalic acid, 2,5-furandicarboxylic acid, and 2,5-pyrroledicarboxylic acid) to form an isoreticular yfm topology. This yields 1D square channels along the c-axis featuring alternating narrow and wide passages with characteristic diameters ranging from ~5.2 to 6.0 Å. CAU-23 exhibits a unique xhh topology due to its 2,5-thiophenedicarboxylate linker. The resulting SBU, incorporating mixed cis-/trans-AlO_6_ octahedra, affords corrugated rhombic channels (~7 Å) along the b-axis. In addition, the heteroatoms within the linkers (O atom in furan, N atom in pyrrole, S atom in thiophene) functionalize the pore walls and fine-tune the local electrostatic environment. Notably, in all these frameworks, the coordination environment of the Al^3+^ is saturated, so there are no open metal sites (OMSs). Obviously, this reasonably designed series provides an ideal platform. Through controllable changes in channel geometry, size, and chemical environment, the synergistic effect of pore confinement and polarity-driven interactions on xenon capture can be systematically studied. This heteroatom engineering strategy for modulating pore polarity in Al-MOFs has been widely explored in previous studies for various separation applications, including hexane isomer [37], xylene [38], acetylene/CO_2_ [39,40], and ethane/ethylene separation [41,42], further supporting the rationality of our material selection.

**Figure 2:**
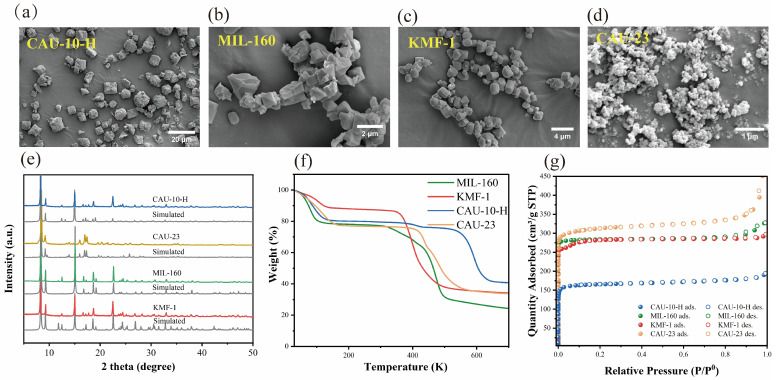
SEM of CAU-10-H (**a**), MIL-160 (**b**), KMF-1 (**c**) and CAU-23 (**d**). (**e**) PXRD pattern of CAU-23, CAU-10-H, MIL-160 and KMF-1. (**f**) The thermogravimetric analyses of CAU-23, MIL-160, KMF-1 and CAU-10-H. (**g**) Nitrogen (N_2_) adsorption–desorption isotherms at 77 K.

The successful synthesis of a series of highly crystalline Al-based MOFs (CAU-10-H, MIL-160, KMF-1, and CAU-23) was established by powder X-ray diffraction (PXRD) (Figure 2e and Figures S8–S11). The excellent agreement between experimental and simulated patterns confirmed their phase purity, while the consistency across the series verified their isostructural nature. The high crystallinity indicated by PXRD was consistent with the observations from scanning electron microscopy (SEM) (Figure 2a–d), where distinct and uniform particulate morphologies were observed. The variations in crystallite size and shape among the four Al-MOFs can be primarily ascribed to differences in synthesis conditions and ligand structures. Specifically, these factors led to distinct crystal growth behaviors under hydrothermal conditions, resulting in the observed morphological diversity. The thermal stability of the MOFs was evaluated by thermogravimetric (TG) analysis. As shown in Figure 2f, all four MOFs exhibit two distinct weight loss steps. The first step, occurring below 150 °C, corresponds to the removal of water and organic solvent molecules from the pore channels. Subsequently, the TG curves of MIL-160, KMF-1, CAU-23, and CAU-10-H display a long plateau, indicating that the materials remain stable within this temperature range without any significant mass loss. The second weight loss step, observed at higher temperatures, signifies the onset of framework decomposition, which begins at approximately 300 °C for MIL-160, 350 °C for KMF-1, and 400 °C for both CAU-23 and CAU-10-H. Beyond these temperatures, a rapid decrease in mass is observed, indicating structural collapse. The microporous nature of four MOFs was confirmed by N_2_ adsorption–desorption measurements at 77 K, which yielded uniform Type-I isotherms (Figure 2g and Figures S1–S4). Their textural properties, however, varied systematically: The Brunauer–Emmett–Teller surface areas (S_BET_) followed the order CAU-10-H (675 m^2^ g^−1^) < KMF-1 (1108 m^2^ g^−1^) < MIL-160 (1195 m^2^ g^−1^) < CAU-23 (1259 m^2^ g^−1^). Correspondingly, the dominant pore sizes, as determined by NLDFT models, increased from approximately 5.2 Å for CAU-10-H and 5.4 Å for KMF-1, to 5.6 Å for MIL-160, and up to ~6.2 Å for CAU-23. The distinct yet systematically varying porosities of these Al-MOFs make them suitable candidate materials for the subsequent evaluation of Xe/Kr separation performance.

### 2.2. Xe/Kr Adsorption and Separation Performance

#### 2.2.1. The Single-Component Adsorption Isotherms of Xe and Kr

**Figure 3:**
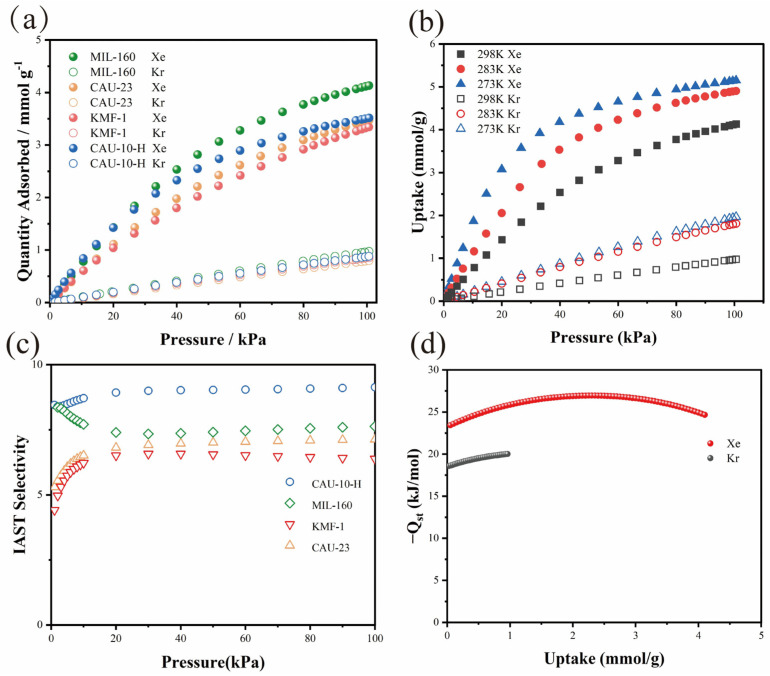
(**a**) Single-component gas adsorption isotherms at 298 K. (**b**) Kr and Xe adsorption isotherms of MIL-160 at 273 K, 283 K and 298 K. (**c**) IAST selectivity of Xe/Kr (20/80, *v*/*v*) at 298 K in CAU-23, MIL-160 and KMF-1. (**d**) Isosteric heat of adsorption (Qst) of Xe and Kr on MIL-160.

The single-component adsorption isotherms of Xe and Kr were measured at 298 K over the pressure range of 0–1 bar. The isotherms revealed that all four MOFs exhibited a preferential uptake of Xe over Kr, which may be due to the larger polarizability and kinetic diameter of Xe (Figure 3a). However, at the practically relevant pressure of 1.0 bar, their Xe uptake capacities clearly diverged. Among them, MIL-160 had the highest Xe absorption at 4.12 mmol g^−1^, outperforming CAU-10-H (3.51 mmol g^−1^), KMF-1 (3.33 mmol g^−1^), and CAU-23 (3.46 mmol g^−1^). As shown in Table S1, the Xe uptake of MIL-160 at 100 kPa reaches 4.12 mmol/g, surpassing almost all previously reported Al-based MOFs, including MIL-120 (1.99 mmol g^−1^) [43], CAU-10-H-CH3-0.21–0.79 (2.87 mmol g^−1^) [33], Al-SDB (1.6 mmol g^−1^) [34], ZUL-C1 (2.88 mmol g^−1^) [31], Al-CDC (2.45 mmol g^−1^) [35], Ag-MOF-303 (3.5 mmol g^−1^) [36] and Al-Fum (3.47 mmol g^−1^) [44]. Its Kr uptake under the same conditions was 0.97 mmol g^−1^, yielding a high Xe/Kr uptake ratio of 4.3. Notably, despite the presence of polar N and S heteroatoms in KMF-1 and CAU-23, respectively, MIL-160 delivered the highest capacity of Xe. This indicates that the optimal balance among pore size, surface area, and polarity is more critical than the mere presence of a polar site. This superior performance is attributed to a synergistic design: The pore aperture (~5.6 Å) provides suitable confinement for Xe (4.047 Å), avoiding severe diffusion limitations; the polar furanyl oxygen atoms enhance dipole interactions; and a high surface area (1195 m^2^ g^−1^) supplies ample adsorption space. Consequently, MIL-160 achieves an optimal compromise between high adsorption capacity and selectivity under near-ambient conditions, which is a critical for efficient separation processes.

Evaluating adsorption at low pressures probes the intrinsic framework–gas affinity, which may reflect adsorption performance in practical, dilute scenarios such as the capture of Xe from nuclear waste streams. At 0.2 bar, CAU-10-H and MIL-160 show virtually identical Xe uptakes of 1.430 and 1.429 mmol g^−1^, respectively, followed by CAU-23 (1.109 mmol g^−1^) and KMF-1 (1.044 mmol g^−1^). The strong performance of CAU-10-H is as expected, arising directly from its smallest pore aperture (~5.2 Å), which provides the strongest spatial confinement for Xe (4.047 Å). Interestingly, MIL-160 rivals similar performance despite its measurably larger pore aperture of ~5.6 Å. The most convincing explanation is that the enhanced polarization provided by the furanyl oxygen atoms effectively counterbalances the less stringent geometric confinement. This aligns with previous reports [37,38,40]. Therefore, this indicates that heteroatom-induced polarization is a powerful factor that enhances Xe affinity, distinct from pore size, which explains how frameworks with non-optimal pore sizes achieve top-level performance under diluted, low-pressure conditions.

Furthermore, to complement the gravimetric data, the volumetric Xe uptake for the four MOFs was also calculated to better evaluate the actual effect of the heteroatom on adsorption performance. As shown in Figure S12, MIL-160 exhibits a volumetric Xe uptake of 102.26 cm^3^ cm^−3^ at 298 K and 100 kPa, which is substantially higher than its Kr uptake (24.18 cm^3^ cm^−3^) under the same conditions. The volumetric Xe capacities of CAU-10-H, KMF-1, and CAU-23 are 88.74, 79.81, and 106.9 cm^3^ cm^−3^, respectively, all of which surpass those of classic MOFs such as [Co_3_(C_4_O_4_)_2_(OH)_2_]·3H_2_O (66.1 cm^3^ cm^−3^) [24], SBMOF-1 (50.9 cm^3^ cm^−3^) [45], CROFOUR-1-Ni (47.1 cm^3^ cm^−3^) [46], and CC3 (53.0 cm^3^ cm^−3^) [47]. Notably, CAU-23 achieves the highest volumetric uptake (106.9 cm^3^ cm^−3^), primarily due to its higher crystal density (1.380 g cm^−3^) and distinct framework topology with larger pore dimensions, indicating that structural factors beyond heteroatom chemistry also play an important role.

#### 2.2.2. Potential of Xe/Kr Separation on MIL-160

To elucidate the host–guest interactions and the specific role of the furanyl oxygen heteroatom, the isosteric heat of adsorption (Qst) for the representative framework MIL-160 was determined from its adsorption isotherms at 273, 283, and 298 K using the virial equation (Figure 3b, Figures S6 and S7). As shown in Figure 3d, the Qst of Xe for MIL-160 is 24.7 kJ mol^−1^, which falls within the optimal range (17.5–35 kJ mol^−1^) [15] and demonstrates a balance between strong adsorption and facile regeneration. This value is notably higher than the Qst for Kr (20.0 kJ mol^−1^). The resulting difference (ΔQst = 4.7 kJ mol^−1^) provides the thermodynamic driving force for the framework’s intrinsic Xe/Kr selectivity. The ideal adsorbed solution theory (IAST) was applied to predict the adsorption selectivity for the Xe/Kr (20:80, *v*/*v*) mixture on MIL-160. (Figure 3c). At 298 K and 1 bar, the selectivity predicted by IAST follow the following order: CAU-10-H (9.13) > MIL-160 (7.63) > CAU-23 (7.13) > KMF-1 (6.38), which is comparable to those of several previously reported Al-based MOFs, such as Al-Fum (8.1) [44] and MIL-120 (9.6) [43]. This trend emphasizes that maximum selectivity can be achieved through precise pore contraction, such as the minimum pore size of CAU-10-H. Importantly, MIL-160 combines the excellent selectivity of 7.6 with its high Xe capacity, thanks to its high Qst of Xe. In summary, the introduction of polar heteroatoms significantly increases the capacity and affinity of Xe, while the selectivity is only moderately sacrificed.

**Figure 4:**
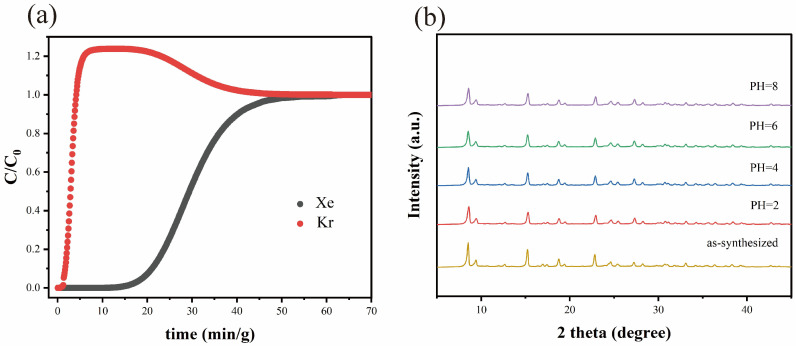
(**a**) Experimental breakthrough curves for a binary Xe/Kr (20:80, *v*/*v*) mixture with a flow rate of 2 mL/min at 298 K and 1.0 bar. (**b**) PXRD patterns of MIL-160 samples soaked in aqueous solution of different pH values for 48 h.

Dynamic fixed-bed breakthrough experiments were carried out at 298 K and 1.0 bar to evaluate the practical Xe/Kr separation performance of MIL-160. A Xe/Kr (20/80, *v*/*v*) gas mixture was introduced into a column packed with 0.4429 g of activated adsorbent at a flow rate of 2 mL·min^−1^. As shown in Figure 4a, Kr eluted rapidly at ~3.0 min g^−1^, while Xe was strongly retained and broke through at a significantly longer time of 42.9 min g^−1^. The long Xe breakthrough time indicates the effective capture and separation of Xe from the mixture by MIL-160. No significant change was observed in the PXRD patterns after the breakthrough experiment, which verifies the robust framework stability of MIL-160 during the dynamic separation process. Moreover, to evaluate the chemical stability of MIL-160, the power was soaked in solutions with pH 2–8 for 48 h. As shown in Figure 4b, the PXRD patterns remained intact, demonstrating that there was no phase change.

### 2.3. Xe/Kr Separation Mechanisms

**Figure 5:**
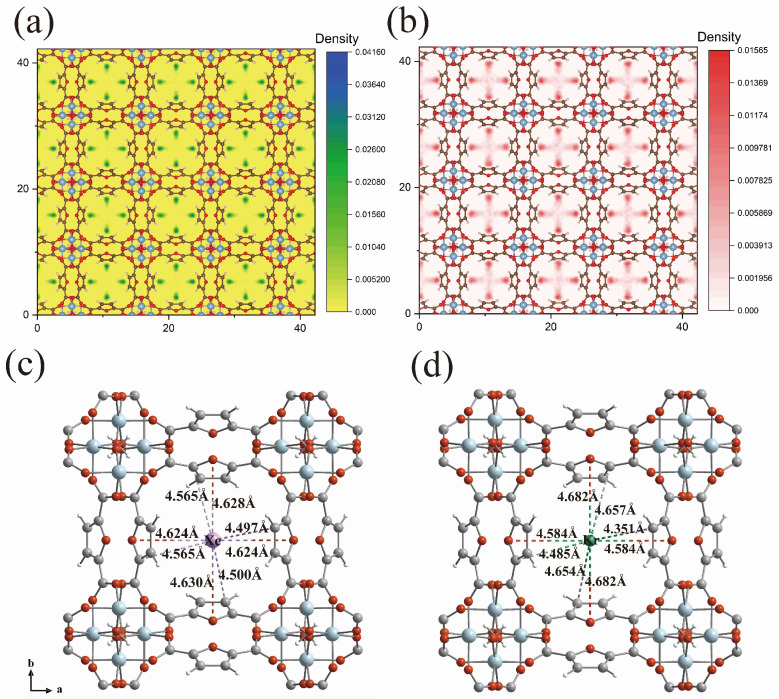
Xe@MOF (**a**) and Kr@MOF (**b**) density distribution calculated by GCMC simulations for MIL-160. (**c**) The DFT-calculated Xe adsorption sites in MIL-160. (**d**) The DFT-calculated Kr adsorption sites in MIL-160. Framework color code: Al, blue; O, red; C, gray; H, white; Xe, purple; Kr, green.

GCMC simulations and DFT-D calculations were conducted with the RASPA 8.1 and CP2K 2.0 packages, respectively, to probe the Xe/Kr separation mechanism of MIL-160. Firstly, GCMC simulations were employed to determine the spatial distribution and primary adsorption sites of Xe and Kr. The density distribution maps at 298 K and 1.0 bar (Figure 5a,b) indicate that the high-density adsorption region of Xe is located within the central pore channel, and the same applies to Kr. It is worth noting that the closest atom to Xe is the furan oxygen atom (4.61 Å, Figure 5c), followed by the carbonyl carbon (4.62 Å). This strongly matched distance combination creates a synergistic effect that facilitates the capture of Xe within the pores. The distribution of adsorption sites for Kr is similar to that of Xe. However, due to its reduced dynamic diameter and polarizability, Kr exhibits a longer average interaction distance. DFT-D calculations provided deeper insights into the interactions of Xe and binding sites. These calculations yielded static binding energies of 49.7 kJ mol^−1^ for Xe and 17.9 kJ mol^−1^ for Kr. Collectively, the simulations confirm that the precise positioning of functional sites is key to the separation performance. This interpretation is supported by the good consistency observed between the theoretical binding energies and the experimental Qst data, validating the concept of pore-environment modulation in Al-MOFs.

## 3. Materials and Methods

### 3.1. Materials

All chemical reagents were commercially obtained and used directly without further purification. Aluminum chloride hexahydrate (AlCl_3_·6H_2_O, 98%), aluminum nitrate nonahydrate (Al(NO_3_)_3_·9H_2_O, 99.99%), and isophthalic acid (C_8_H_6_O_4_, 99%) were purchased from Adamas Reagent Co., Ltd. (Shanghai, China). Aluminum sulfate octadecahydrate (Al_2_(SO_4_)_3_·18H_2_O, 99.95%) and thiophene-2,5-dicarboxylic acid (C_6_H_4_O_4_S, 98%) were supplied by Innochem Science and Technology Co., Ltd. (Beijing, China). Sodium metaaluminate (NaAlO_2_, technical grade) and 2,5-furandicarboxylic acid (C_6_H_4_O_5_, 97%) were obtained from Shanghai Aladdin Biochemical Technology Co., Ltd. (Shanghai, China). 1H-Pyrrole-2,5-dicarboxylic acid (C_6_H_5_NO_4_, 98%) was purchased from Beijing LeYan Chemical Co., Ltd. (Beijing, China). N, N-Dimethylformamide (DMF, 99%) and ethanol absolute (99.7%) were supplied by Sinopharm Chemical Reagent Co., Ltd. (Shanghai, China). Ultrapure water was prepared using a laboratory water purification system from Merck KGaA, Darmstadt, Germany.

### 3.2. Synthesis

MIL-160 was synthesized according to a reported literature procedure with some modifications [38]. A total of 0.1603 g NaOH (4.008 mmol) and 0.3123 g 2,5-furandicarboxylic acid (2.00 mmol) were added to a round-bottomed flask (50 mL) containing 10 mL distilled water and the mixture was stirred until clear. Then, 0.4886 g AlCl_3_·6H_2_O (2.024 mmol) was added to the clear solution. The mixture was transferred to a 50 mL Teflon-lined steel autoclave and heated at 100 °C for 12 h in a convection oven. The white precipitate was separated by using filtration and purified with deionized water and ethanol. Finally, the obtained solid was dried overnight at 100 °C in the oven. Before adsorption analyses, the sample was activated at 150 °C for 10 h under reduced pressure condition.

CAU-10-H was synthesized according to a reported literature procedure with some modifications [48]. A mixture of Al(NO_3_)_3_·9H_2_O (2.00 g, 5.331 mmol), isophthalic acid (1.00 g, 6.019 mmol), H_2_O (18 mL) and DMF (5 mL) was placed in a 50 mL Teflon-lined steel autoclave, heated to 135 °C and held for 12 h in a convection oven. Then, the mixture was cooled to room temperature and the white precipitate was separated by filtration and purified with deionized water and ethanol. Finally, the obtained solid was dried overnight at 100 °C in the oven. Prior to adsorption analyses, the sample was activated at 150 °C for 10 h under reduced pressure conditions.

KMF-1 was synthesized according to a previously reported procedure [41]. Al_2_(SO_4_)_3_·18H_2_O (2.148 g, 3.223 mmol) was dissolved into 17.5 mL deionized water to afford a clear solution, which was then slowly added to a mixture of 2,5-pyrroledicarboxylic acid (1.00 g, 6.447 mmol), NaOH (0.645 g, 16.12 mmol) and deionized water (17.5 mL). After stirring at room temperature for 30 min, the reaction solution was heated to reflux for 12 h. Then, the white precipitate was separated by using filtration and purified with deionized water and ethanol. Finally, the obtained solid was dried overnight at 100 °C in the oven. Prior to adsorption analyses, the sample was activated at 150 °C for 10 h under reduced pressure conditions.

CAU-23 was synthesized according to a reported literature procedure with some modifications [49]. A total of 0.43 g thiophene-2,5-dicarboxylic acid (2.50 mmol) was stirred with 0.20 g sodium hydroxide (5.00 mmol) in 10 mL distilled water until a clear solution of Na_2_TDC was obtained. Then, 1.875 mL of aqueous aluminum chloride solution (1 mol/L, 1.875 mmol) and 1.250 mL of aqueous sodium aluminate solution (0.5 mol/L, 0.625 mmol) was added. The mixture was placed in a 25 mL Teflon-lined steel autoclave, heated to 105 °C and held for 12 h in a convection oven. Then, the white precipitate was separated by using filtration and purified with deionized water and ethanol. Finally, the obtained solid was dried overnight at 100 °C in the oven. Prior to adsorption analyses, the sample was activated at 150 °C for 10 h under reduced pressure conditions.

### 3.3. Characterization

Powder X-ray diffraction (PXRD) was collected on a Rigaku Smart Lab SE (Neu-Isenburg, Germany, Cu Kα radiation, λ = 1.54056 Å). The thermogravimetric analyses (TGAs) were performed on a NETZSCH TG209 F3 system in nitrogen and under 1 atm of pressure at a heating rate of 10 °C min^−1^. The nitrogen adsorption and desorption isotherms at 77 K were carried out on a BSD-660M A3M. Single-component Xe and Kr adsorption isotherms were performed by using a Micromeritics 3Flex adsorption apparatus (Norcross, GA, USA). Breakthrough measurements were performed using a BSD-MAB Analyzer (Beijing, China). Ultrahigh-purity grade N_2_ (99.999%), Kr (99.999%), and Xe (99.999%) were applied for all measurements.

### 3.4. Fitting of Single-Component Adsorption Isotherms

The adsorption isotherms for Xe and Kr in MIL-160 were measured at 273 K, 283 K, and 298 K, while for Xe and Kr in CAU-23, CAU-10-H and KMF-1, the isotherms were collected at 298 K. The pure component isotherms were fitted using the Dual-site Langmuir–Freundlich (DSLF) model, depicted as follows:

(1) $$N=A_{1}\frac{b_{1}P^{C_{1}}}{1+b_{1}P^{C_{1}}}+A_{2}\frac{b_{2}P^{C_{2}}}{1+b_{2}P^{C_{2}}}$$

where A_1_ and A_2_ (mmol·g^−1^) are the saturated capacities of site 1 and site 2, b_1_ and b_2_ (1·kPa^−1^) are the affinity coefficients to the sites 1 and 2, P (kPa) is the pressure of the bulk gas at equilibrium with the adsorbed phase (kPa), N (mmol·g^−1^) is the gas uptake amount of an adsorbent, and C_1_ and C_2_ represent the deviations from an ideal homogeneous surface. The fitting parameters of the DSLF equation are listed in Table S1.

### 3.5. IAST Calculations

Ideal adsorbed solution theory (IAST) has been used to predict a wide range of binary gas separation performances. By fitting the pure gas adsorption isotherms of gas components, the IAST selectivity can be calculated according to the equation defined as follows:

(2) $$S=\frac{x_{1}/x_{2}}{y_{1}/y_{2}}$$

where $x_{i}$ is the molar fraction of gas 1 and gas 2 in the adsorbed phase and $y_{i}$ is the molar fraction of gas 1 and gas 2 in the bulk phase.

### 3.6. Calculation of Isosteric Heat of Adsorption

A virial-type equation comprising the temperature-independent parameters *a_i_* and *b_j_* was employed to calculate the enthalpies of adsorption for Xe (273 K, 283 K and 298 K) and Kr (273 K, 283 K and 298 K) on MIL-160, respectively. In each case, the data were fitted using the following equation:

(3) $$\ln P=\ln N+1/T\sum_{i=0}^{m}a_{i}N_{i}+\sum_{j=0}^{n}b_{j}N_{j}$$

Here, *P* is the pressure in mmHg, *N* is the amount adsorbed in mmol/g, T represents the temperature in K, *a_i_* and *b_j_* are virial coefficients, and m, n represents the number of virial coefficients required to adequately describe the isotherms (the number of the virial coefficient was determined by gradually increasing m and n until the contribution of adding extra m and n was deemed to be statistically insignificant to the overall fit. And the average value of squared deviations from the experiment values was minimized). Then, the isosteric heat of adsorption for Xe, Kr on MIL-160 can be calculated using the following equation:

(4) $$Q_{st}=-R\sum_{i=0}^{m}a_{i}N_{i}$$

Q_st_ is the coverage-dependent isosteric heat of adsorption and R represents the universal gas constant.

### 3.7. Theoretical Calculations

Density functional theory (DFT) calculations were performed using CP2K (version 8.1) [50] to optimize the MOF structure and compute adsorption energies of Kr and Xe with MOF. Geometry optimization employed the Quickstep module with a Gaussian and Plane Waves (GPWs) method in CP2K code. The Perdew–Burke–Ernzerhof (PBE) exchange–correlation functional [51] was used with Grimme’s DFT-D3 dispersion correction (including C9 terms) to account for van der Waals interactions [52]. A double-ζ valence-polarized (DZVP-MOLOPT-SR-GTH) basis set and GTH-PBE pseudopotentials were applied to all elements (C, H, O, Al, Kr, Xe) [53]. The plane-wave cutoff was set to 400 Ry with a 60 Ry relative cutoff. A 1×1×2 Monkhorst–Pack k-point grid sampled the Brillouin zone. Self-consistent field (SCF) convergence was set to $1\times 10^{-6}$ Ha with a maximum of 2000 iterations. Analytical stress tensor calculations enabled full cell optimization. Adsorption energies ($E_{\mathrm{ads}}$) were computed as $E_{\mathrm{ads}}=E_{\mathrm{MOF}+\mathrm{gas}}-(E_{\mathrm{MOF}}+E_{\mathrm{gas}})$, where $E_{\mathrm{MOF}+\mathrm{gas}}$ is the energy of the gas-adsorbed MOF, $E_{\mathrm{MOF}}$ is the optimized MOF energy, and $E_{\mathrm{gas}}$ is the energy of an isolated gas molecule in a large box.

Grand Canonical Monte Carlo (GCMC) simulations were executed using RASPA 2.0 [54] to model Kr and Xe adsorption at 298.15 K and 1 bar. The DFT-optimized MOF structure was used as a rigid framework. Following optimization, RESP atomic partial charges were derived at the same level as the DFT calculations [50]. Simulations employed 2×2×3 supercell with periodic boundaries. TraPPE force fields described gas molecules, while UFF parameters modeled MOF–gas interactions [54]. Electrostatic interactions were calculated via Ewald summation with a 12 Å cutoff [55]. Each simulation ran $2\times 10^{7}$ steps (50% equilibration, 50% production) to ensure convergence. Adsorption densities and spatial distributions were extracted from ensemble averages.

## 4. Conclusions

This study systematically investigated the influence of heteroatom-induced polarization on Xe adsorption and Xe/Kr separation within a series of robust aluminum-based metal–organic frameworks (Al-MOFs) sharing common one-dimensional channel structures. By employing a designed platform of isoreticular and topologically distinct Al-MOFs functionalized with N, O, and S heteroatoms, we have demonstrated that the incorporation of polar sites, exemplified by the furanyl oxygen in MIL-160, significantly enhances Xe uptake capacity and framework affinity. Notably, MIL-160 achieved an optimal balance, exhibiting the highest working capacity under ambient conditions coupled with effective dynamic separation performance. A key mechanistic insight revealed that the enhanced polarization from the heteroatom can compensate for less stringent geometric confinement, as evidenced by the comparable low-pressure Xe uptake between MIL-160 and the smallest pore analog CAU-10-H. Combined experimental and computational analyses confirmed that this superior performance stems from a synergistic microenvironment where suitable pore dimensions and heteroatom-facilitated, induced dipole interactions cooperatively enhance Xe trapping. Collectively, this work establishes that the precise synergy between pore confinement and chemical polarity, rather than the mere presence of a polar site, is the critical determinant for efficient Xe/Kr separation in stable MOFs, providing a fundamental principle for the rational design of advanced adsorbents.

## Data Availability

Data are contained within the article.

## Supplementary Materials

The following supporting information can be downloaded at: https://www.mdpi.com/article/10.3390/molecules31050891/s1, Table S1. Summary of the uptake, selectivities and dynamic Xe capacity for Xe and Kr in various materials; Table S2. Dual-site Langmuir-Freundlich parameter fits for Xe and Kr in Al-MOFs; Figure S1. N_2_ adsorption isotherms at 77 K and PSD plot of activated CAU-23; Figure S2. N_2_ adsorption isotherms at 77 K and PSD plot of activated MIL-160; Figure S3. N_2_ adsorption isotherms at 77 K and PSD plot of activated KMF-1; Figure S4. N_2_ adsorption isotherms at 77 K and PSD plot of activated CAU-10-H; Figure S5. Xe and Kr sorption isotherms for (a) MIL-160, (b) CAU-23, (c) KMF-1, and (d) CAU-10-H at 298 K; Figure S6. Virial fitting of the Xe adsorption isotherms for MIL-160; Figure S7. Virial fitting of the Kr adsorption isotherms for MIL-160; Figure S8. Powder X-ray diffraction patterns of CAU-10-H; Figure S9. Powder X-ray diffraction patterns of MIL-160; Figure S10. Powder X-ray diffraction patterns of KMF-1; Figure S11. Powder X-ray diffraction patterns of CAU-23; Figure S12. Single-component gas adsorption isotherms at 298 K for CAU-10-H, MIL-160, KMF-1 and CAU-23. References [24,31,33,34,35,36,43,44,45,46,47,56,57,58,59,60,61,62,63,64,65,66,67,68,69,70] are cited in the Supplemental Materials.
