# Supplementary material for: Heteroatom Engineering in Robust Al-Based MOFs for Efficient Separation of Xenon over Krypton
Source: Molecules. 2026 Mar 7;31(5):891. doi: 10.3390/molecules31050891 (PMC12985661; doi:10.3390/molecules31050891)
Supplement: Supplementary file 1 [file molecules-31-00891-s001.zip › molecules-4151917-supplementary.pdf]

Supplementary Information

Table S1. Summary of the uptake, selectivities and dynamic Xe capacity for Xe and Kr in various materials

Sample	Xe uptake (mmol g^{-1}) 20 kPa	Xe uptake (mmol g^{-1}) 100 kPa	IAST selectivity (20/80) 298 K	Ref
MIL-160	1.43	4.12	7.63	This work
CAU-23	1.11	3.46	7.13	This work
KMF-1	1.04	3.33	6.38	This work
CAU-10-H	1.43	3.50	9.13	This work
ZU-62	0.20	3.76	8.1	[1]
HOF-BTB	0.49	2.01	6	[2]
SIFSIX-3-Ni	0.54	2.51	5.5	[3]
NbOFFIVE-Cu-TPA	0.60	2.63	5.1	[4]
GeFSIX-Cu-TPA	0.67	2.73	5.3	[4]
ZU-66 (ZrFSIX-2-Zn-i)	0.69	3.18	6.7	[1]
TIFSIX-Cu-TPA	0.74	2.88	5.4	[4]
LPC-MOF	0.80	1.30	11	[5]
ECUT-50	0.90	2.26	8	[6]
UTSA-74	0.90	2.70	8.4	[7]
$Ni(4\text{-}DPDS)_2MoO_4$	0.91	1.07	20.2	[8]
PAF-45S	0.95	1.85	24.7	[9]
SIFSIX-3-Cu	0.98	2.11	4.8	[10]
$Ni(4\text{-}DPDS)_2WO_4$	1.00	1.11	30.2	[8]
ZU-42 (ZrFSIX-2-Cu-i)	1.02	2.68	5.5	[1]
NU-200	N.A.	4.91	9.3	[11]
NU-403	N.A.	1.2	9	[12]
NU-1107-Ag(I)	0.92	1.56	13.4	[13]
ZJU-HOF-6a	N.A.	3.3	23.5	[14]
MIL-120	1.42	1.99	9.6	[15]
CAU-10-H-CH3-0.21-0.79	1.29	2.87	10.00	[16]
Al-SDB	1.0	1.6	13.8	[17]
ZUL-C1	1.76	2.88	11.7	[18]
ZUL-C2	2.05	2.58	19.1	[18]
Al-CDC	N.A.	2.45	10.7	[19]
Ag-MOF-303	1.83	3.5	10.4	[20]
Al-Fum	1.52	3.47	8.1	[21]
Al-Fum-Me	1.75	3.01	10.0	[21]
Co-squarate	1.18	1.35	69.7	[22]
SBMOF-1	1.27	1.47	16	[23]
SBMOF-2	1.46	2.85	10	[24]

CROFOUR-1-Ni	1.11	1.79	22	[25]
CROFOUR-2-Ni	0.84	1.5	15.5	[25]
CC3	1.66	2.44	20.4c	[26]

Table S2. Dual-site Langmuir-Freundlich parameter fits for Xe and Kr in Al-MOFs. The fits are based on experimental isotherm data at 298 K.

Adsorbates	Materials	A_1	b_1	C_1	A_2	b_2	C_2	R^2
Xe	MIL-160	5.120	0.004	1.424	0.372	0.157	1.185	0.999
	CAU-23	5.660	0.006	1.174	0.064	0.278	1.839	0.999
	KMF-1	6.651	0.005	1.127	0.296	0.103	1.225	0.999
	CAU-10-H	3.842	0.005	1.408	0.620	0.123	1.125	0.999
Kr	MIL-160	7.039	0.00029	1.233	1.070	0.008	0.936	0.999
	CAU-23	5.047	0.00083	1.159	0.055	0.038	1.324	0.999
	KMF-1	13.291	0.00075	0.978	0.008	0.000006	3.792	0.999
	CAU-10-H	4.035	0.0011	1.166	0.098	0.034	1.230	0.999

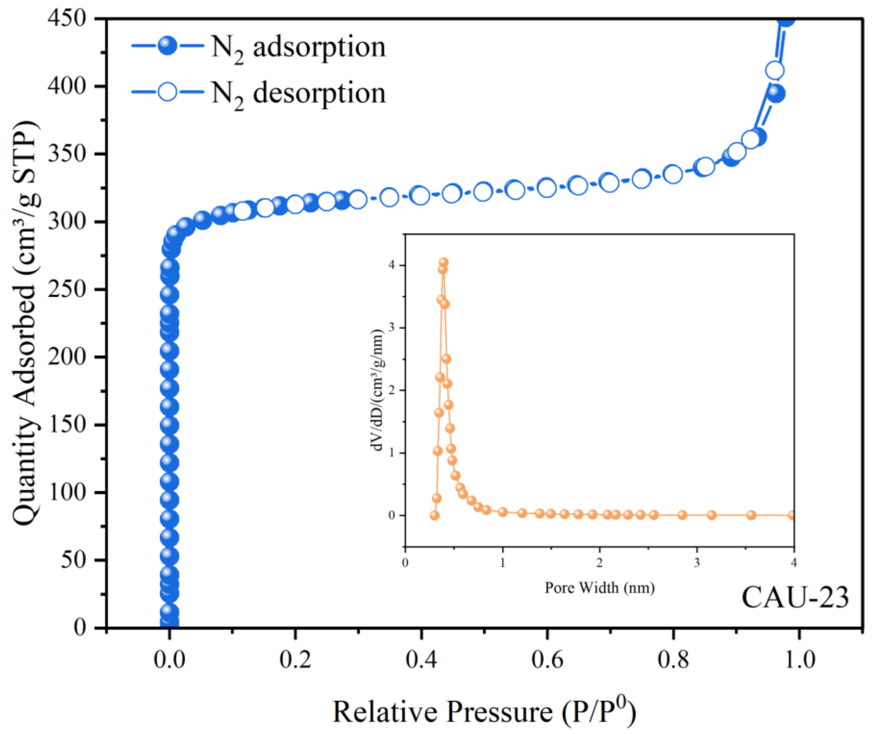

Figure S1. N_2 adsorption isotherms at 77 K and PSD plot of activated CAU-23.

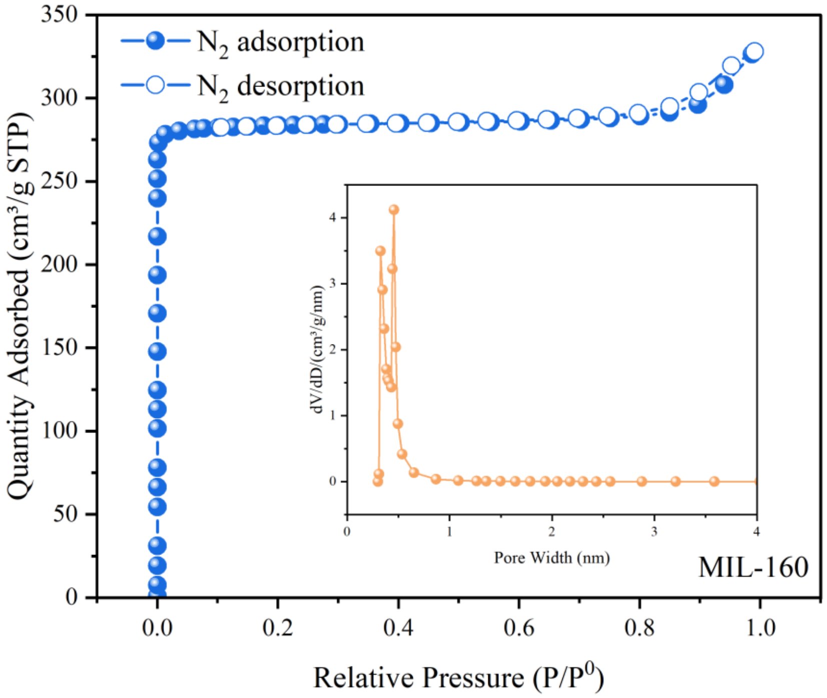

Figure S2. N_2 adsorption isotherms at 77 K and PSD plot of activated MIL-160

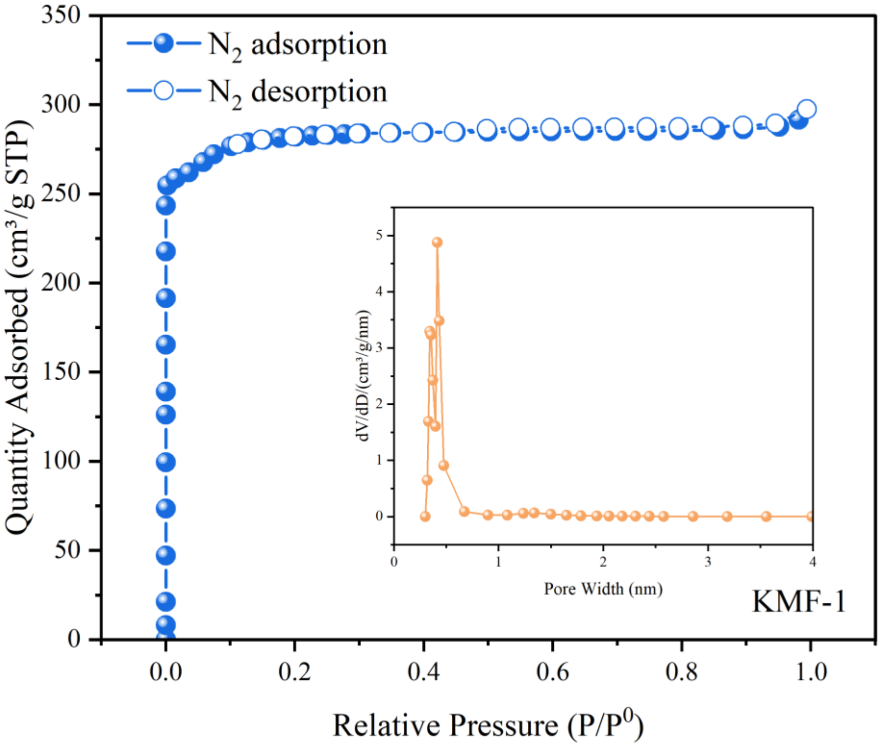

Figure S3. N_2 adsorption isotherms at 77 K and PSD plot of activated KMF-1.

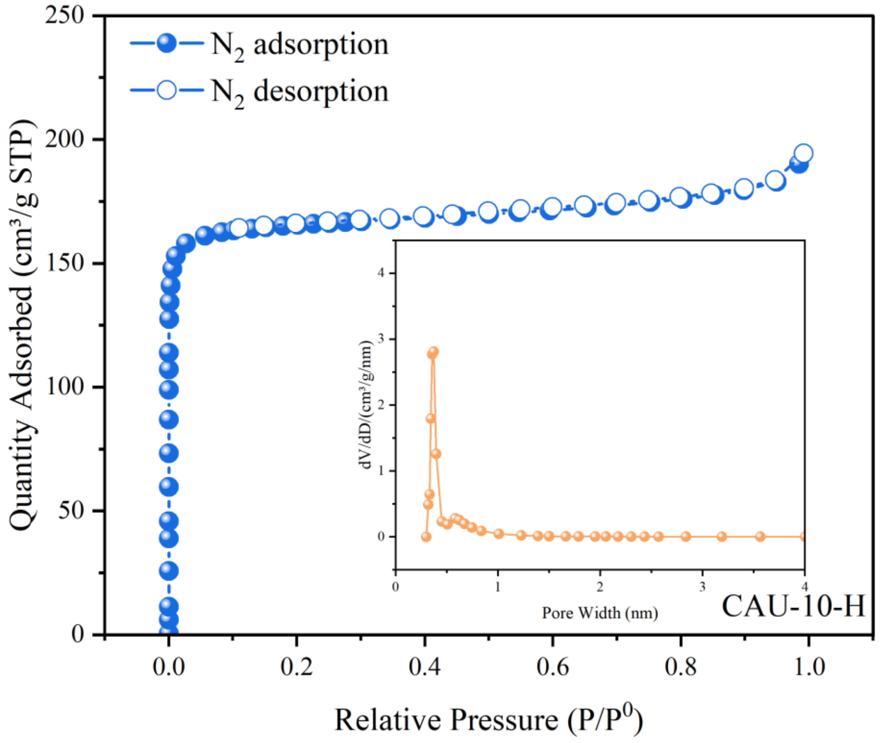

Figure S4. N_2 adsorption isotherms at 77 K and PSD plot of activated CAU-10-H.

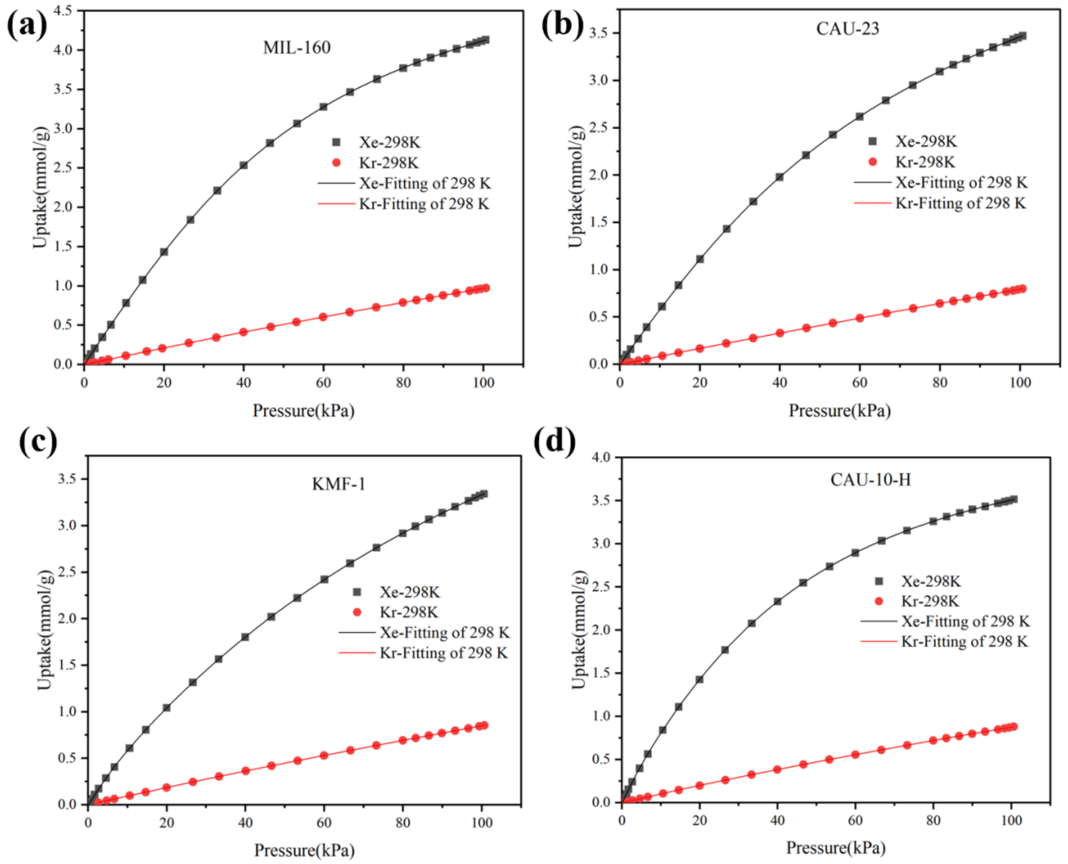

Figure S5. Xe and Kr sorption isotherms for (a) MIL-160, (b) CAU-23, (c) KMF-1, and (d) CAU-10-H at 298 K.

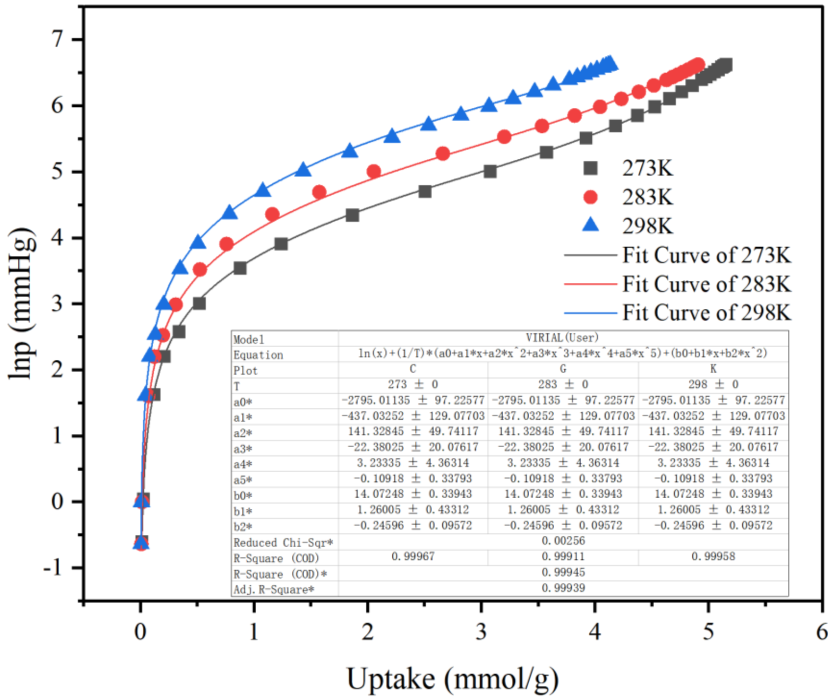

Model	VIRIAL(User)		
Equation	ln(x)+(1/T)*(a0+a1*x+a2*x^2+a3*x^3+a4*x^4+a5*x^5)+(b0+b1*x+b2*x^2)		
Plot	C	G	K
T	273 ± 0	283 ± 0	298 ± 0
a0*	-2795.01135 ± 97.22577	-2795.01135 ± 97.22577	-2795.01135 ± 97.22577
a1*	-437.03252 ± 129.07703	-437.03252 ± 129.07703	-437.03252 ± 129.07703
a2*	141.32845 ± 49.74117	141.32845 ± 49.74117	141.32845 ± 49.74117
a3*	-22.38025 ± 20.07617	-22.38025 ± 20.07617	-22.38025 ± 20.07617
a4*	3.23335 ± 4.36314	3.23335 ± 4.36314	3.23335 ± 4.36314
a5*	-0.10918 ± 0.33793	-0.10918 ± 0.33793	-0.10918 ± 0.33793
b0*	14.07248 ± 0.33943	14.07248 ± 0.33943	14.07248 ± 0.33943
b1*	1.26005 ± 0.43312	1.26005 ± 0.43312	1.26005 ± 0.43312
b2*	-0.24596 ± 0.09572	-0.24596 ± 0.09572	-0.24596 ± 0.09572
Reduced Chi-Sqr*	0.00256		
R-Square (COD)	0.99967	0.99911	0.99958
R-Square (COD)*	0.99945		
Adj. R-Square*	0.99939		

Figure S6. Virial fitting of the Xe adsorption isotherms for MIL-160.

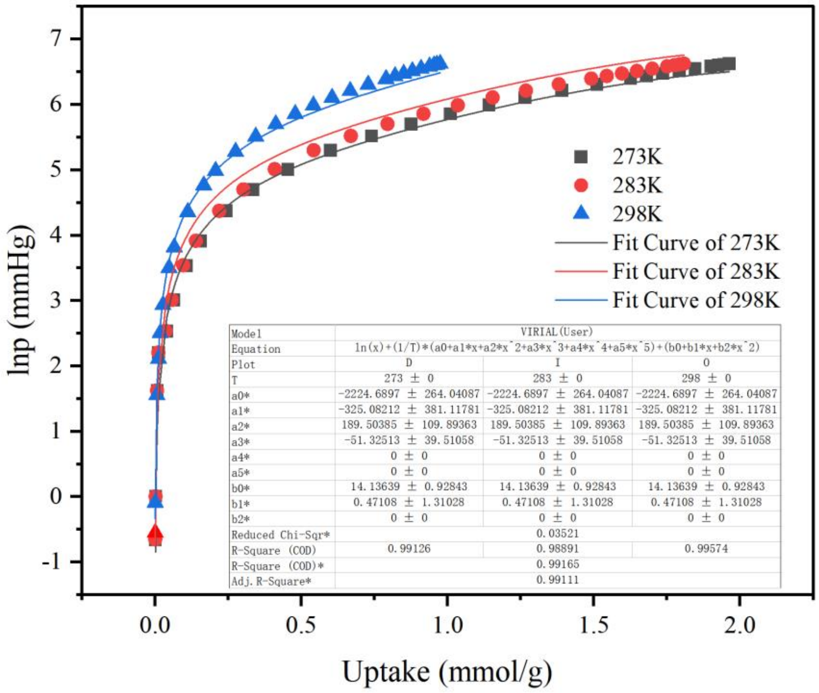

Figure S7. Virial fitting of the Kr adsorption isotherms for MIL-160.

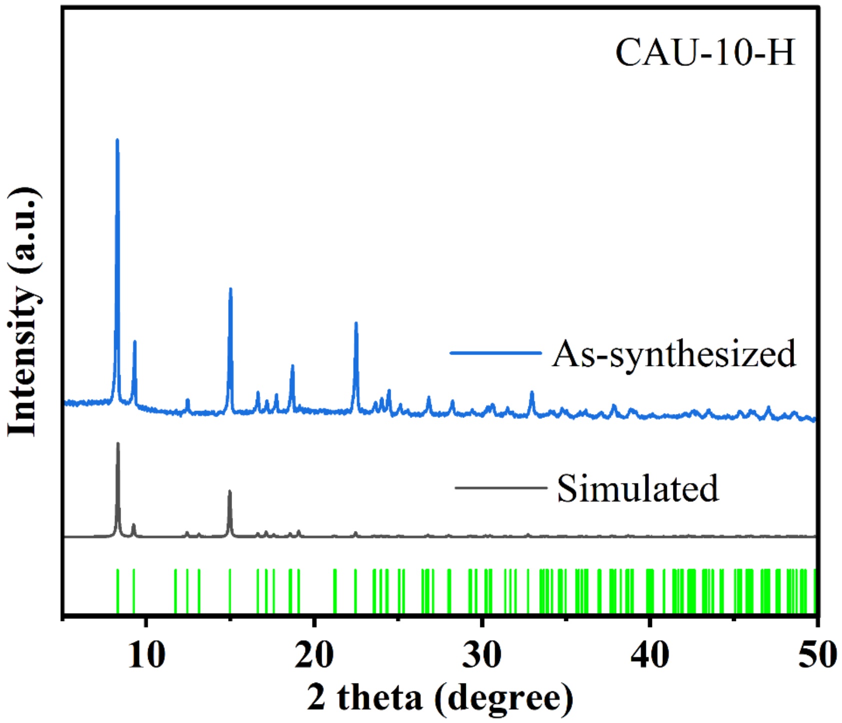

Figure S8. Powder X-ray diffraction patterns of CAU-10-H.

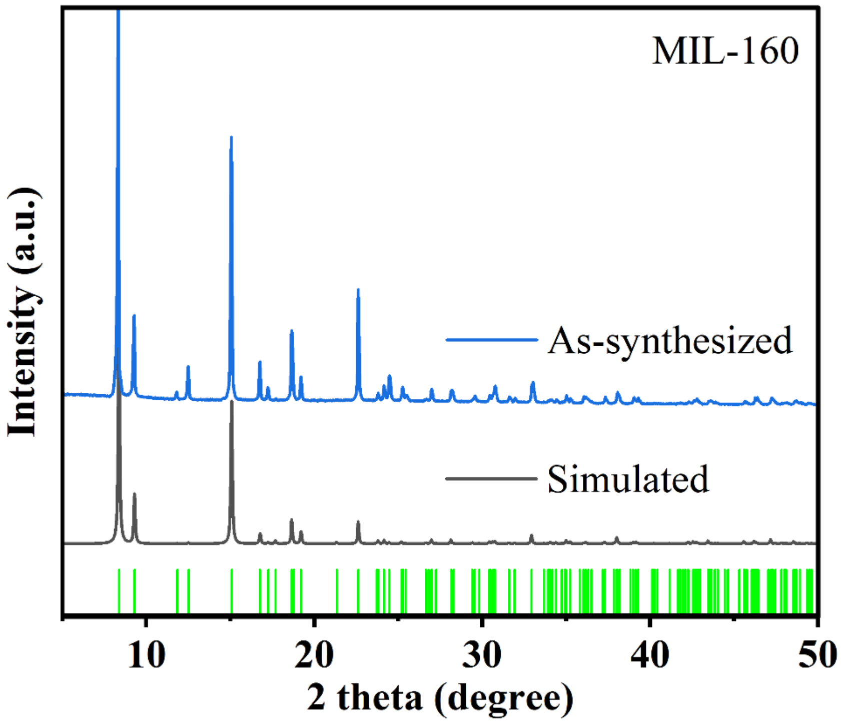

Figure S9. Powder X-ray diffraction patterns of MIL-160.

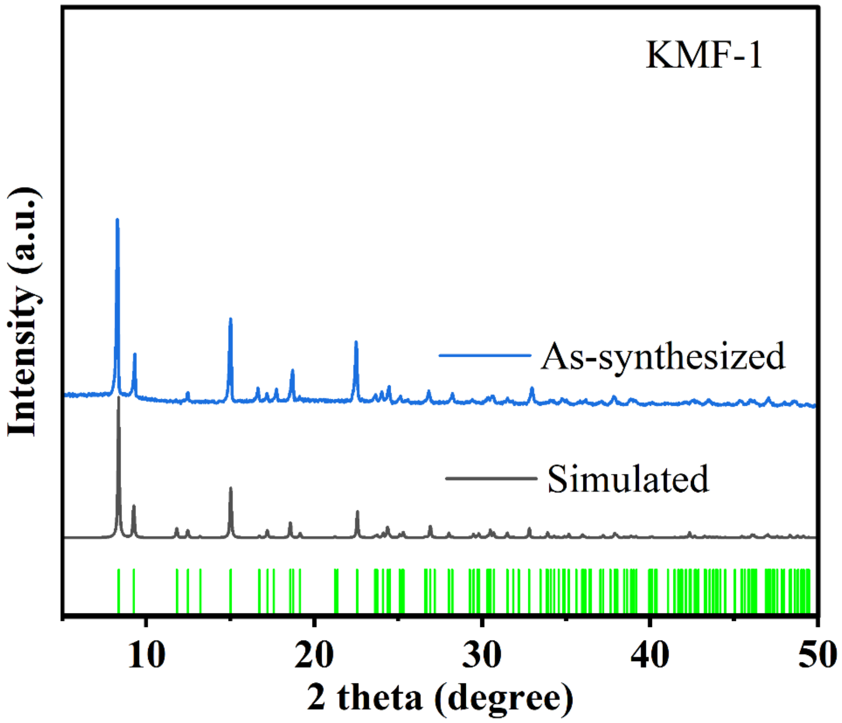

Figure S10. Powder X-ray diffraction patterns of KMF-1.

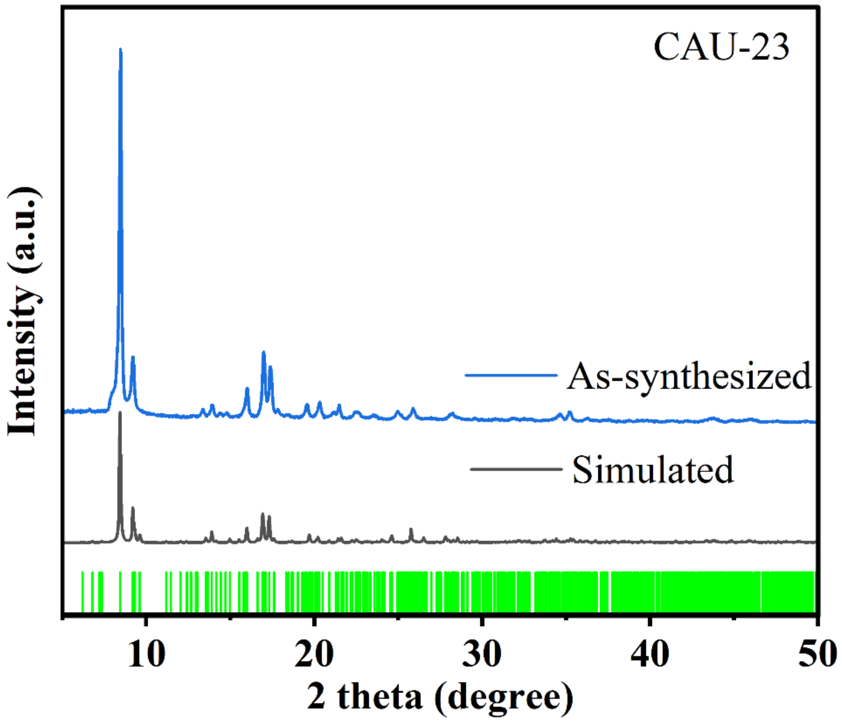

Figure S11. Powder X-ray diffraction patterns of CAU-23.

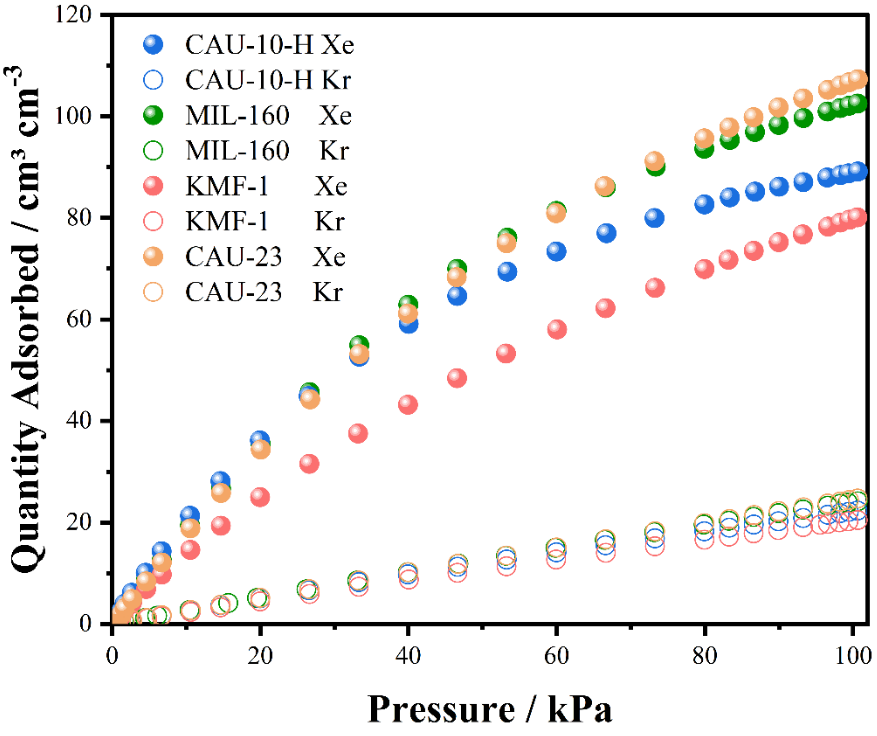

Figure S12. Single-component gas adsorption isotherms at 298 K for CAU-10-H, MIL-160, KMF-1 and CAU-23.

Reference

1. Wang, Q.; Ke, T.; Yang, L.; Zhang, Z.; Cui, X.; Bao, Z.; Ren, Q.; Yang, Q.; Xing, H. Separation of Xe from Kr with Record Selectivity and Productivity in Anion-Pillared Ultramicroporous Materials by Inverse Size-Sieving. *Angew Chem Int Ed* **2020**, *59*, 3423–3428, doi:10.1002/anie.201913245.
2. Lee, W.-G.; Yoon, T.-U.; Bae, Y.-S.; Kim, K.S.; Baek, S.B. Selective Separation of Xe/Kr and Adsorption of Water in a Microporous Hydrogen-Bonded Organic Framework. *RSC Adv.* **2019**, *9*, 36808–36814, doi:10.1039/C9RA08184D.
3. Elsaidi, S.K.; Mohamed, M.H.; Simon, C.M.; Braun, E.; Pham, T.; Forrest, K.A.; Xu, W.; Banerjee, D.; Space, B.; Zaworotko, M.J.; et al. Effect of Ring Rotation upon Gas Adsorption in SIFSIX-3-M (M = Fe, Ni) Pillared Square Grid Networks. *Chem. Sci.* **2017**, *8*, 2373–2380, doi:10.1039/C6SC05012C.
4. Li, J.; Wang, L.; Chen, Y.; Gu, Z.; Jiang, T.; Luan, B.; Krishna, R.; Zhang, Y. Efficient Xe/Kr Separation in Fluorinated Pillar-Caged Metal-Organic Frameworks. *Microporous and Mesoporous Materials* **2023**, *357*, 112631, doi:10.1016/j.micromeso.2023.112631.
5. Wang, X.; Ma, F.; Xiong, S.; Bai, Z.; Zhang, Y.; Li, G.; Chen, J.; Yuan, M.; Wang, Y.; Dai, X.; et al. Efficient Xe/Kr Separation Based on a Lanthanide–Organic Framework with One-Dimensional Local Positively Charged Rhomboid

Channels. *ACS Appl. Mater. Interfaces* **2022**, *14*, 22233–22241, doi:10.1021/acsami.2c05258.
6. Wang, L.; Liu, W.; Ding, J.; Zhang, H.; Zhu, Y.; Luo, F. A Robust Calcium–Organic Framework for Effective Separation of Xenon and Krypton. *Crystal Growth & Design* **2021**, *21*, 954–959, doi:10.1021/acs.cgd.0c01302.
7. Tao, Y.; Fan, Y.; Xu, Z.; Feng, X.; Krishna, R.; Luo, F. Boosting Selective Adsorption of Xe over Kr by Double-Accessible Open-Metal Site in Metal–Organic Framework: Experimental and Theoretical Research. *Inorg. Chem.* **2020**, *59*, 11793–11800, doi:10.1021/acs.inorgchem.0c01766.
8. Zheng, F.; Guo, L.; Chen, R.; Chen, L.; Zhang, Z.; Yang, Q.; Yang, Y.; Su, B.; Ren, Q.; Bao, Z. Shell-like Xenon Nano-Traps within Angular Anion-Pillared Layered Porous Materials for Boosting Xe/Kr Separation. *Angew Chem Int Ed* **2022**, *61*, e202116686, doi:10.1002/anie.202116686.
9. Li, J.; Huang, L.; Zou, X.; Zheng, A.; Li, H.; Rong, H.; Zhu, G. Porous Organic Materials with Ultra-Small Pores and Sulfonic Functionality for Xenon Capture with Exceptional Selectivity. *J. Mater. Chem. A* **2018**, *6*, 11163–11168, doi:10.1039/C8TA03991G.
10. Elsaidi, S.K.; Mohamed, M.H.; Helal, A.S.; Galanek, M.; Pham, T.; Suepaul, S.; Space, B.; Hopkinson, D.; Thallapally, P.K.; Li, J. Radiation-Resistant Metal-Organic Framework Enables Efficient Separation of Krypton Fission Gas from Spent Nuclear Fuel. *Nat Commun* **2020**, *11*, 3103, doi:10.1038/s41467-020-16647-1.
11. Gong, W.; Xie, Y.; Pham, T.; Shetty, S.; Son, F.; Idrees, K.; Chen, Z.; Xie, H.; Liu, Y.; Snurr, R.; et al. Creating Optimal Pockets in a Clathrochelate-Based Metal-Organic Framework for Gas Adsorption and Separation: Experimental and Computational Studies. *Journal of the American Chemical Society* **2022**, *144*, 3737–3745, doi:10.1021/jacs.2c00011.
12. Idrees, K.B.; Chen, Z.; Zhang, X.; Mian, M.R.; Drout, R.J.; Islamoglu, T.; Farha, O.K. Tailoring Pore Aperture and Structural Defects in Zirconium-Based Metal–Organic Frameworks for Krypton/Xenon Separation. *Chem. Mater.* **2020**, *32*, 3776–3782, doi:10.1021/acs.chemmater.9b05048.
13. Gong, W.; Xie, Y.; Wang, X.; Kirlikovali, K.O.; Idrees, K.B.; Sha, F.; Xie, H.; Liu, Y.; Chen, B.; Cui, Y.; et al. Programmed Polarizability Engineering in a Cyclen-Based Cubic Zr(IV) Metal–Organic Framework to Boost Xe/Kr Separation. *J. Am. Chem. Soc.* **2023**, *145*, 2679–2689, doi:10.1021/jacs.2c13171.
14. Wang, J.-X.; Xiong, S.; Gu, X.-W.; Wen, H.-M.; Zhang, X.; Chen, B.; Li, B.; Qian, G. Channel-Pocket Ultra-Micropore Configuration in a Hydrogen-Bonded Organic Framework for Simultaneous High Xe Capture and Xe/Kr Separation. *Angew. Chem. Int. Ed.* **2025**, *64*, e202515759, doi:10.1002/anie.202515759.
15. Zhang, P.; Zhong, Y.; Yao, Q.; Liu, X.; Zhang, Y.; Wang, J.; Deng, Q.; Zeng, Z.; Deng, S. Robust Ultramicroporous Metal–Organic Framework with Rich Hydroxyl-Decorated Channel Walls for Highly Selective Noble Gas Separation. *J. Chem. Eng. Data* **2020**, *65*, 4018–4023, doi:10.1021/acs.jced.0c00323.
16. Liu, B.; Yan, Z.; Liu, Q.; Gong, Y.; Wu, X.; Mao, Z.; Xiong, S.; Hu, S. Boosting

Xe/Kr Separation by a Mixed-Linker Strategy in Radiation-Resistant Aluminum-Based Metal − Organic Frameworks. *Separation and Purification Technology* **2023**, *311*, 123335, doi:10.1016/j.seppur.2023.123335.
17. Kim, M.; Robinson, A.; Sushko, M.; Thallapally, P. Aluminum-Based Microporous Metal-Organic Framework for Noble Gas Separation. *Journal of Industrial and Engineering Chemistry* **2023**, *118*, 181–186, doi:10.1016/j.jiec.2022.11.003.
18. Zhou, J.; Ke, T.; Steinke, F.; Stock, N.; Zhang, Z.; Bao, Z.; He, X.; Ren, Q.; Yang, Q. Tunable Confined Aliphatic Pore Environment in Robust Metal–Organic Frameworks for Efficient Separation of Gases with a Similar Structure. *J. Am. Chem. Soc.* **2022**, *144*, 14322–14329, doi:10.1021/jacs.2c05448.
19. Zhu, Z.; Li, B.; Liu, X.; Zhang, P.; Chen, S.; Deng, Q.; Zeng, Z.; Wang, J.; Deng, S. Efficient Xe/Kr Separation on Two Metal-Organic Frameworks with Distinct Pore Shapes. *Separation and Purification Technology* **2021**, *274*, 119132, doi:10.1016/j.seppur.2021.119132.
20. Wang, H.; Shi, Z.; Yang, J.; Sun, T.; Rungtaweevoranit, B.; Lyu, H.; Zhang, Y.; Yaghi, O.M. Docking of Cu^{I} and Ag^{I} in Metal–Organic Frameworks for Adsorption and Separation of Xenon. *Angew Chem Int Ed* **2021**, *60*, 3417–3421, doi:10.1002/anie.202015262.
21. Yan, Z.; Gong, Y.; Yang, C.; Wu, X.; Liu, B.; Liu, Q.; Xiong, S.; Peng, S. Pore Size Reduction by Methyl Function in Aluminum-Based Metal- Organic Frameworks for Xenon/Krypton Separation. *Crystal Growth & Design* **2020**, *20*, 8039–8046, doi:10.1021/acs.cgd.0c01283.
22. Li, L.; Guo, L.; Zhang, Z.; Yang, Q.; Yang, Y.; Bao, Z.; Ren, Q.; Li, J. A Robust Squarate-Based Metal–Organic Framework Demonstrates Record-High Affinity and Selectivity for Xenon over Krypton. *J. Am. Chem. Soc.* **2019**, *141*, 9358–9364, doi:10.1021/jacs.9b03422.
23. Banerjee, D.; Simon, C.M.; Plonka, A.M.; Motkuri, R.K.; Liu, J.; Chen, X.; Smit, B.; Parise, J.B.; Haranczyk, M.; Thallapally, P.K. Metal–Organic Framework with Optimally Selective Xenon Adsorption and Separation. *Nat Commun* **2016**, *7*, ncomms11831, doi:10.1038/ncomms11831.
24. Chen, X.; Plonka, A.M.; Banerjee, D.; Krishna, R.; Schaef, H.T.; Ghose, S.; Thallapally, P.K.; Parise, J.B. Direct Observation of Xe and Kr Adsorption in a Xe-Selective Microporous Metal–Organic Framework. *J. Am. Chem. Soc.* **2015**, *137*, 7007–7010, doi:10.1021/jacs.5b02556.
25. Mohamed, M.H.; Elsaidi, S.K.; Pham, T.; Forrest, K.A.; Schaef, H.T.; Hogan, A.; Wojtas, L.; Xu, W.; Space, B.; Zaworotko, M.J.; et al. Hybrid Ultra-Microporous Materials for Selective Xenon Adsorption and Separation. *Angew Chem Int Ed* **2016**, *55*, 8285–8289, doi:10.1002/anie.201602287.
26. Chen, L.; Reiss, P.S.; Chong, S.Y.; Holden, D.; Jelfs, K.E.; Hasell, T.; Little, M.A.; Kewley, A.; Briggs, M.E.; Stephenson, A.; et al. Separation of Rare Gases and Chiral Molecules by Selective Binding in Porous Organic Cages. *Nature Mater* **2014**, *13*, 954–960, doi:10.1038/nmat4035.
